# The First-Reported Presentation of Quadruple Locations of Elastofibroma Dorsi: A Case Report and Review of the Literature

**DOI:** 10.7759/cureus.41425

**Published:** 2023-07-05

**Authors:** April Ngoy, Konstantin Tchalukov, Gabriel Pollock, Bryon Thomson, Christopher Nguyen

**Affiliations:** 1 Department of Radiology, Kaweah Health, Visalia, USA; 2 Department of Radiology, Riverside University Health System Medical Center, Moreno Valley, USA

**Keywords:** msk radiology, mri images, soft tissue masses, benign tumor, elastofibroma dorsi

## Abstract

Elastofibroma dorsi (EFD) is an uncommon benign tumor of mesenchymal origin that usually occurs in the subscapular region. Bilateral and triple EFD are frequently reported in the literature but cases with more lesions have never been described. Our patient is a 50-year-old female with quadruple locations of bilateral suprascapular and subscapular EFD who presented with left shoulder pain and swelling over the affected area. Clinical presentation, computed tomography (CT) and magnetic resonance imaging (MRI), and biopsy were consistent with EFD. Therapeutic excision was performed and successfully alleviated the patient’s discomfort. This report presents the first case of quadruple locations of EFD and highlights the value of MRI in the diagnosis of EFD, especially when there are multiple masses with indistinct margins are deeply located in the chest wall.

## Introduction

Elastofibroma dorsi (EFD) is a rare benign tumor of mesenchymal origin that most commonly occurs in middle-aged women and in the elderly [1]. Due to its unspecific clinical presentation, it may be difficult to make an accurate diagnosis based on clinical signs and symptoms alone. Medical imaging plays an important role in the diagnosis of EFD and in the detection of additional, often bilateral, masses. In this case report, we present quadruple locations of suprascapular and subscapular EFD in a 50-year-old woman with progressively worsening left shoulder pain. Our objective is to highlight the diagnostic importance of MRI in identifying EFD, particularly in the case of quadruple locations, which has never been reported previously.

## Case presentation

**Figure 1:**
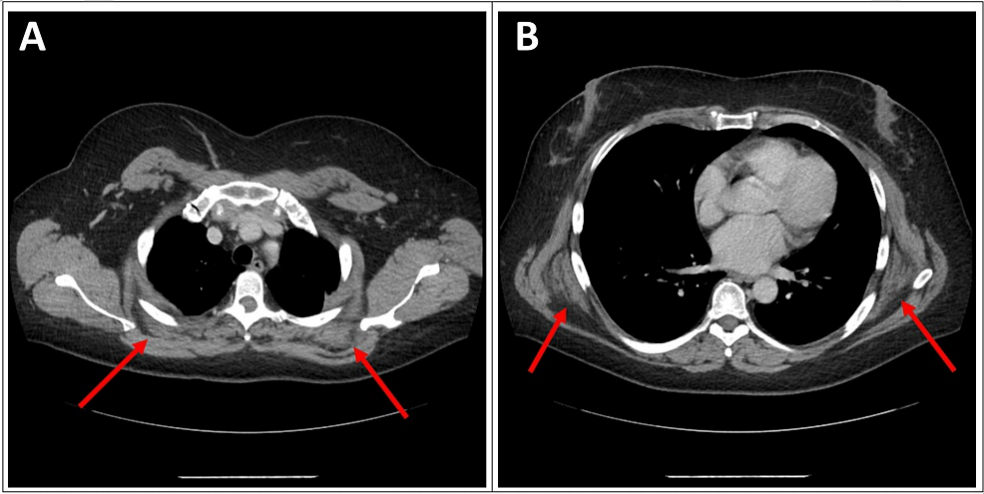
Contrast-enhanced axial plane CT images demonstrate (A) bilateral suprascapular soft tissue density masses, located medial to the medial scapular border and deep to the trapezius muscles and (B) bilateral subscapular masses located deep to the serratus anterior and latissimus dorsi muscles.  The masses contain alternating soft tissue and fat density components.

A 50-year-old female without a significant medical history presented to the orthopedic clinic for non-radiating, 7/10 aching left shoulder pain, and nontender localized swelling over the left scapular area. She reported the swelling has been present for three years and has not changed in size over time. She works as a custodian and has missed work recently due to pain that is worse at the end of her shift. The pain improved with ibuprofen, ice, and rest but shortly returned with shoulder movement. Physical examination revealed a firm, mobile mass on the superomedial border of the left scapula. The pain was most notable when the shoulder was abducted >75° and when the mass was palpated. A computed tomography (CT) scan demonstrated bilateral suprascapular soft tissue density masses located medial to the medial scapular border and deep to the trapezius muscles, as well as bilateral subscapular masses located deep to the serratus anterior and latissimus dorsi muscles (Figures 1A, 1B).

**Figure 2:**
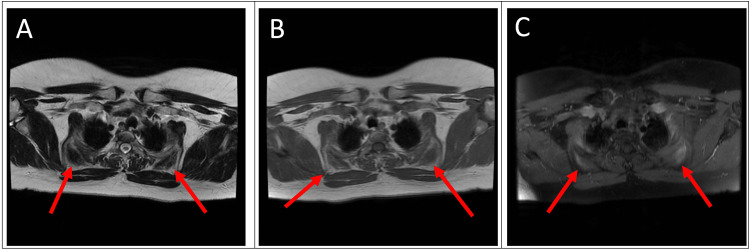
Axial (A) T2-weighted MRI reveals alternating soft tissue and fatty components of bilateral suprascapular masses. (B) T1-weighted MRI of bilateral suprascapular masses containing alternating soft tissue and fatty components. (C) T1-weighted post-contrast with fat saturation image showing low-level enhancement.

**Figure 3:**
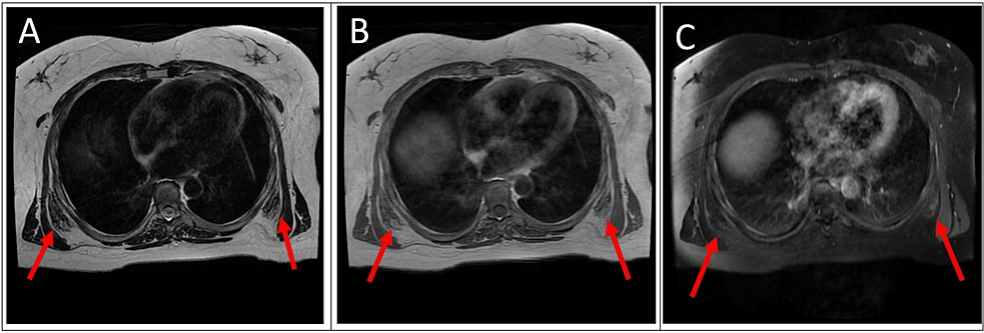
Axial (A) T2-weighted MRI reveals alternating soft tissue and fatty components of bilateral subscapular masses. Axial (B) T1-weighted MRI of bilateral suprascapular masses containing alternating soft tissue and fatty components. (C) T1-weighted post-contrast image with fat saturation showing low-level enhancement.

**Figure 4:**
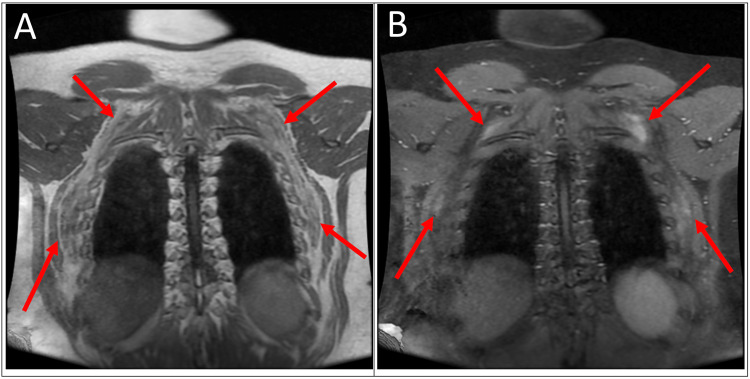
(A) Coronal T1-weighted image demonstrates bilateral suprascapular and bilateral subscapular masses. The masses appear to contain alternating soft tissue and fatty components. (B) T1-weighted post-contrast image with fat saturation showing low-level enhancement.

Magnetic resonance imaging (MRI) was performed to further characterize the masses. The MR examination showed ill-defined bilateral suprascapular and bilateral subscapular masses with alternating soft tissue and fatty components on T1- and T2- weighted imaging (Figures 2A-2C, 3A-3C, 4A-4B). The lesions were heterogenous, striated, and showed low-level enhancement. The imaging findings of the lesions were characteristic of EFD.

**Figure 5:**
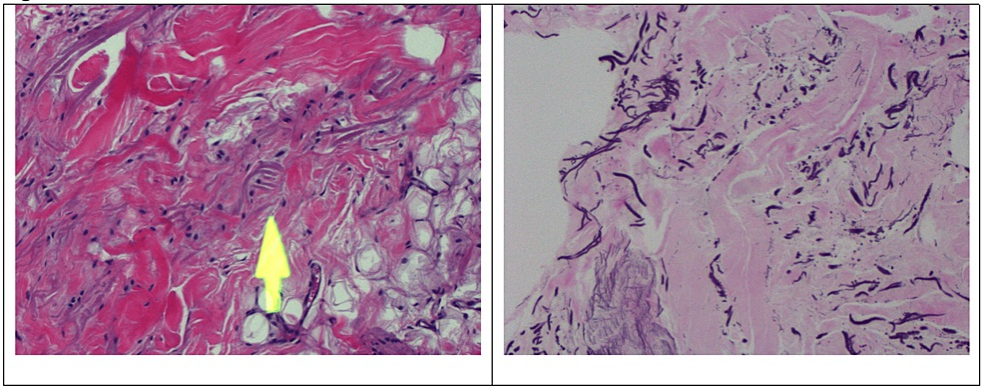
(A) H&E stain at 100X magnification shows a mixture of dense collagenous stroma, bland fibroblasts, and mature adipose tissue. Some dystrophic elastic fibers are seen (yellow arrow). (B) Hart elastic special stain at 100X magnification highlights the presence of dystrophic elastic fibers.

Given the effect on her quality of life, the patient elected for arthroscopic surgical excision of the left suprascapular mass. Perioperatively, a biopsy of the left suprascapular mass was performed, and pathology was consistent with EFD (Figures 5A, 5B). There were no immediate postoperative complications. On a follow-up visit three weeks after the surgery, the patient reported a pain score of <2/10 compared to 7/10, which was present preoperatively with shoulder movement. On physical exam, an active full range of motion of the left shoulder was seen, whereas pain limited shoulder abduction to <75° preoperatively. She was able to return to work with minimal shoulder discomfort.

## Discussion

To our knowledge, the presented case is the first with quadruple locations of EFD ever reported. Its clinical presentation, imaging appearance, and locations are typical of EFD. EFD typically grows beneath the rhomboid major and latissimus dorsi muscles adjacent to the inferior angle of the scapula, which account for 93% of cases [2]. Unusual locations of EFD include the ischial tuberosity, olecranon area, deltoid muscle, axilla, intraspinal space, and greater omentum [3,4]. EFD usually is unilateral and right-sided but can be bilateral in 10-66% of cases [2,5].

The pathogenesis of EFD is unclear and still a matter of debate. One study suggests a familial predisposition due to an underlying enzymatic defect [4,6]. In a study based in Okinawa Prefecture, Japan, it was noted that 32% of the reported cases occurred within a single family [6]. However, many authors believe the mass is caused by friction of the lower scapula against the thoracic wall due to repetitive minor trauma and manual labor [7]. In addition, vascular insufficiency, elastotic degradation of collagen, and abnormal elastotic fibrinogenesis may account for its pathogenesis [7].

EFD is slow growing and often asymptomatic and thus can remain undetected clinically. When EFD is symptomatic, pain is the most common presenting chief complaint, with presentation ranging from mild to severely disabling pain. Due to the unspecific clinical presentation of EFD, medical imaging is important in delineating it from other, more aggressive soft tissue tumors of the chest wall such as sarcoma, fibroma, hemangioma, desmoid tumor, and aggressive fibromatosis [4]. Indication for surgery depends on the severity of symptoms and, most importantly, patient preference [8]. Our patient’s primary complaint was left shoulder pain that prevented her from being able to fulfill the demands of her job. The surgical treatment successfully allowed her to return to work. However, our case is limited due to the loss of follow-up after the three-week postoperative check. We were unable to assess for the recurrence of symptoms or delayed postoperative complications.

MRI is the modality of choice for diagnosing EFD. On MRI, it is usually a semilunar-shaped mass, heterogenous, low to iso-signal intensity compared to skeletal muscle with high-signal intensity streaks in parallel to the longest axis both on T1- and T2-weighted images, similar to that of skeletal muscle [9,10]. CT, like MRI, shows a heterogenous mass with indistinct margins and an attenuation similar to that of muscle [9]. Diffusion-weighted magnetic resonance imaging (DWI) represents a promising new diagnostic tool for EFD, providing more specific findings like functional information about the diffusivity of water molecules and highlighting high cellularity lesions throughout the body [5]. A case series based in Istanbul that included 10 patients showed that EFD can demonstrate marked diffusion restriction on DWI [5]. While the diagnosis of EFD can often be made with imaging alone, a core needle biopsy may be completed to characterize the histology of equivocal soft tissue lesions [8]. Although biopsy is the most definitive method to correctly diagnose the tumor, on occasion, the sparse cellularity makes it difficult to interpret the cytology [11]. Tumor histopathology characteristics include the identification of collagen as well as coarse and enlarged elastic fibers [11].

## Conclusions

Although elastofibroma dorsi is usually known to be unilateral, reported cases of bilateral lesions are not uncommon. Therefore, when they are detected on one side, further investigation should be performed to assess for bilateral lesions. In our patient, imaging detected the first reported case of double bilateral lesions. Our case also highlights that when multiple lesions have poorly defined margins that fixate deeply on muscles, MRI findings help distinguish this benign diagnosis from other, more aggressive soft tissue lesions.
